# Genomic Metrics Applied to *Rhizobiales* (*Hyphomicrobiales*): Species Reclassification, Identification of Unauthentic Genomes and False Type Strains

**DOI:** 10.3389/fmicb.2021.614957

**Published:** 2021-03-25

**Authors:** Camila Gazolla Volpiano, Fernando Hayashi Sant’Anna, Adriana Ambrosini, Jackson Freitas Brilhante de São José, Anelise Beneduzi, William B. Whitman, Emanuel Maltempi de Souza, Bruno Brito Lisboa, Luciano Kayser Vargas, Luciane Maria Pereira Passaglia

**Affiliations:** ^1^Departamento de Genética, Instituto de Biociências, Universidade Federal do Rio Grande do Sul, Porto Alegre, Brazil; ^2^Departamento de Diagnóstico e Pesquisa Agropecuária, Secretaria Estadual da Agricultura, Pecuária e Desenvolvimento Rural, Porto Alegre, Brazil; ^3^Department of Microbiology, University of Georgia, Athens, GA, United States; ^4^Departamento de Bioquímica e Biologia Molecular, Centro Politécnico, Setor de Ciências Biológicas, Universidade Federal do Paraná, Curitiba, Brazil

**Keywords:** ANI, dDDH, species-cluster, genome clustering, *Rhizobium*

## Abstract

Taxonomic decisions within the order *Rhizobiales* have relied heavily on the interpretations of highly conserved 16S rRNA sequences and DNA–DNA hybridizations (DDH). Currently, bacterial species are defined as including strains that present 95–96% of average nucleotide identity (ANI) and 70% of digital DDH (dDDH). Thus, ANI values from 520 genome sequences of type strains from species of *Rhizobiales* order were computed. From the resulting 270,400 comparisons, a ≥95% cut-off was used to extract high identity genome clusters through enumerating maximal cliques. Coupling this graph-based approach with dDDH from clusters of interest, it was found that: (i) there are synonymy between *Aminobacter lissarensis* and *Aminobacter carboxidus*, *Aurantimonas manganoxydans* and *Aurantimonas coralicida*, “*Bartonella mastomydis*,” and *Bartonella elizabethae*, *Chelativorans oligotrophicus*, and *Chelativorans multitrophicus*, *Rhizobium azibense*, and *Rhizobium gallicum*, *Rhizobium fabae*, and *Rhizobium pisi*, and *Rhodoplanes piscinae* and *Rhodoplanes serenus*; (ii) *Chelatobacter heintzii* is not a synonym of *Aminobacter aminovorans*; (iii) “*Bartonella vinsonii*” subsp. *arupensis* and “*B. vinsonii*” subsp. *berkhoffii* represent members of different species; (iv) the genome accessions GCF_003024615.1 (“*Mesorhizobium loti* LMG 6125^T^”), GCF_003024595.1 (“*Mesorhizobium plurifarium* LMG 11892^T^”), GCF_003096615.1 (“*Methylobacterium organophilum* DSM 760^T^”), and GCF_000373025.1 (“*R. gallicum* R-602 sp^T^”) are not from the genuine type strains used for the respective species descriptions; and v) “*Xanthobacter autotrophicus*” Py2 and “*Aminobacter aminovorans*” KCTC 2477^T^ represent cases of misuse of the term “type strain”. *Aminobacter heintzii* comb. nov. and the reclassification of *Aminobacter ciceronei* as *A. heintzii* is also proposed. To facilitate the downstream analysis of large ANI matrices, we introduce here ProKlust (“Prokaryotic Clusters”), an R package that uses a graph-based approach to obtain, filter, and visualize clusters on identity/similarity matrices, with settable cut-off points and the possibility of multiple matrices entries.

## Introduction

The classification of *Rhizobiales* species is complex and has undergone many changes over the years. Frank (1889) described *Rhizobium*, the type genus of the order, to accommodate different symbiotic nitrogen-fixing bacteria based only on their selective interaction with legume plants. In 1991, this classification was abandoned after extensive criticism (Wilson, 1944) and the discovery that genes required for symbiosis are often located on transmissible plasmids (Nuti et al., 1979; Brewin et al., 1980; Prakash et al., 1981). The minimal standards for species descriptions have usually incorporated DNA–DNA hybridization (DDH) and the 16S rRNA sequence analyses, as well as morphological, physiological, and biochemical features (Graham et al., 1991).

In the pre-genomic era, the DDH was considered the gold standard for prokaryotic species circumscriptions. The DDH measures the fraction of DNA that hybridizes under optimal conditions, and a threshold of at least 70% between two strains was widely recognized as the species boundary (Tindall et al., 2010). In the first formal species definition based on the DDH proposed by Wayne et al. (1987), the change in the melting temperature (ΔTm) was recommended as a second measure of genetic relatedness to be considered with DDH, and a ΔTm value of 5°C or less was established as the cut-off for prokaryotic species. The ΔTm represents the difference of the Tm of the heteroduplex of the two strains being tested, reflecting primarily the sequence identity (Johnson and Whitman, 2007). However, because DDH is technically easier to measure, ΔTm is usually not determined in most species’ descriptions (Li et al., 2015).

With the advent of genomics, taxonomy has relied heavily on comparative measurements that calculate surrogates of both the ΔTm and DDH *in silico* directly between genomes sequences. These comparisons, as forms of similarity or distance, have been coined as overall genome-related indices (OGRI). Among them, average nucleotide identity (ANI) using the BLASTn algorithm to perform alignments (ANIb) and the Genome BLAST Distance Phylogeny (GBDP)-based digital DDH (dDDH) methods have been most widely used (Konstantinidis and Tiedje, 2005; Richter and Rosselló-Móra, 2009; Chun et al., 2018; Sant’Anna et al., 2019). The ANI is considered a good surrogate for the ΔTm because it only compares homologous DNA fragments that meet sequence identity and coverage criteria. In its turn, the dDDH using formula *d0* and *d6* seems to most closely approximate the properties expected for the experimentally determined DDH values (Auch et al., 2010a; Li et al., 2015).

The ANIb was originally proposed on the basis of benchmarking with respect to DDH values by Goris et al. (2007). In this method, the genomic sequence from one of the genomes in a pair (“query”) is cut *in silico* into consecutive 1,020 nt fragments, mimicking the DNA fragmentation step during the DDH experiments. The fragments are then used to search against the whole genomic sequence of the other genome in the pair (“reference”) using BLASTn. The ANI between the query and the reference genomes is calculated as the mean identity of all BLASTn matches that show more than 30% overall sequence identity (recalculated to an identity along the entire sequence) over an alignable region of at least 70% of their length. The classical cut-off point of 70% DDH for species delineation corresponded to an ANIb of 95%. Later, Richter and Rosselló-Móra (2009) recommended applying an ANI boundary of 95–96% after correlating ANIm (ANI with MUMmer ultra-rapid aligning tool) and DDH values between strains putatively representing single species. In its turn, Meier-Kolthoff et al. (2013) established a 70% dDDH for species boundaries based on the comparisons of the dDDH predictions with *in vitro* DDH.

The application of OGRI to prokaryote taxonomy has the advantage of being objective and proved to unravel a microbial phylogenetic novelty at an unprecedented pace (Sant’Anna et al., 2019; Sanford et al., 2021), however, it is still being subject to controversies. The ANI and dDDH cut-offs were calibrated to yield the species previously determined by the DDH and ΔTm, which were based on a threshold that was calibrated upon previous bacterial species definitions that were based entirely on phenotypic properties, mostly in the *Enterobacteriaceae* (Gevers et al., 2006). Finally, some authors consider that a universal metric or species cut-off for *in silico* genome comparisons should be interpreted only as a guideline considering that all species have evolved via independent evolutionary trajectories which could result in cohesion among individuals occurring at different levels of similarity (Palmer et al., 2020).

In the analysis of genomic sequence data from prokaryotes with OGRI values, new algorithms with optimized search parameters (Lee I. et al., 2016; Rodriguez-R and Konstantinidis, 2016) or to speeding up the search process (Richter and Rosselló-Móra, 2009; Varghese et al., 2015; Yoon et al., 2017b; Jain et al., 2018) are continuously being developed. However, effective algorithms for mining the output data from large OGRI matrices are still in high demand. A diverse set of “traditional” hierarchical clustering approaches (i.e., average linkage, complete linkage, Ward, UPGMA, and neighbor-joining) are already available. These approaches can create clusters by grouping genes/genomes with high identity measures together and returning tree-shaped diagrams. Such diagrams do not allow overlapping clusters and are difficult to extract information in complex cases where not all the members of a cluster share sufficient identity to cluster with all the other members. Moreover, it is usually not possible to combine different matrices in such approaches. This is important considering that ANI values should be used in conjunction with complementary measures of the minimum amount that genomes must overlap, such as the alignment coverage (pyANI), AF (Alignment Fraction; Varghese et al., 2015), or dDDH. If the homologous regions are short with respect to the total length of the genomes, as might be seen following an horizontal gene transfer (HGT), then ANI values may be high even though the bacteria are distantly related.

The present study aims to evaluate the taxonomic structure at the species level within the *Rhizobiales* order using genome-scale comparisons with ANIb and dDDH from a collection of 520 genome assemblies identified as belonging to type strains. We note, however, that Hördt et al. (2020) recently proposed an emended description of *Hyphomicrobiales* (Douglas, 1957) to replace *Rhizobiales* (Kuykendall, 2005). Our secondary objective is to introduce ProKlust, an R package developed to facilitate the downstream analysis of large identity matrices.

## Materials and Methods

### Organisms, Culturing Media, and DNA Extraction

*Ensifer terangae* SEMIA 6460^T^ (USDA 4894^T^ = ORS 1009^T^ = LMG 7834^T^ = ATCC 51692^T^ = DSM 11282^T^) and Rhizobium gallicum SEMIA 4085^T^ (USDA 2918^T^ = R-602 sp^T^ = EMBRAPA Soja 172^T^), previously isolated from common bean nodules and maintained at SEMIA Culture Collection (World Data Center on Microorganisms no. 443), were re-hydrated from lyophilized cultures and grown on yeast mannitol (YM) agar medium (Somasegaran and Hoben, 2012) at 28°C. DNA from late log phase SEMIA 6460^T^ cultures was extracted using the PureLink^TM^ Microbiome DNA Purification Kit (Thermo Fisher Scientific). A phenol: chloroform method adapted from Sambrook and Russell (2006) was used to obtain the genomic DNA from SEMIA 4085^T^.

### Sequencing and *de novo* Genome Assembly

Genomic Encyclopedia of Bacteria and Archaea (GEBA) KMG Phase III project from the United States Department of Energy (DOE) Joint Genome Institute (JGI) provided a draft genome sequence for SEMIA 6460^T^. Genomic libraries for SEMIA 4085^T^ were prepared at the Department of Biochemistry and Molecular Biology (UFPR, Brazil) using the Nextera prep kit (Illumina). The sequencing was performed on an Illumina MiSeq platform with a 250 paired-end protocol. SPAdes v.3.11.1 (Bankevich et al., 2012) was used to assemble the reads, and Blobtools (Laetsch and Blaxter, 2017) was used to identify and remove contaminated contigs.

### Download of Public Genome Sequence Data

Genomic sequences for 518 assemblies were retrieved from the NCBI assembly database upon searching for “*Rhizobiales*” with filters “latest RefSeq,” and “assembly from any type” on April 29th, 2020. Additional 21 assemblies were retrieved from the database upon searching for “*Aminobacter*” with filters “latest RefSeq” on November 26th, 2020.

### Genomics Metrics Computation

Pairwise comparisons among the genomes were calculated using the ANIb method from pyANI v 0.2.10 Python3 module^1^ and FastANI v 1.3 (Jain et al., 2018). GGDC (Genome-to-Genome Distance Calculator) 2.1 with the recommended BLAST+ aligner were used to compute dDDH values and confidence intervals (C.I.) using GBDP formula *d0* (GGDC formula 1) and *d6* (GGDC formula 3) at http://ggdc.dsmz.de.

### ProKlust Development and Usage

ProKlust uses a graph-based approach implemented in R language for the downstream analysis of large identity/similarity matrices. First, the input pairwise matrix is formatted into a triangular matrix using the average of each pair. Then, the matrix is formatted using the cut-off values chosen by the user. To obtain the Boolean matrix, values were replaced according to the criterion chosen. If more than one matrix is used as input, the generated logical matrices are also multiplied to obtain a consensus. Afterward, the graph is formed by connecting the nodes (i.e., genomes or genes) using a modified Bron–Kerbosch algorithm from the “igraph” R package (Csardi and Nepusz, 2006) to find the maximal cliques, which is superior in performance (Eppstein et al., 2010). The following filters were implemented to filter the data (i) “filterRemoveIsolated,” to remove isolated nodes i.g., nodes that do not form groups/clusters; (ii) “filterRemoveLargerComponent” and “filterOnlyLargerComponent” to remove or retain only the component containing the highest number of nodes); (iii) “filterDifferentNamesConnected” to retain groups of connected nodes containing more than one binomial species name; and (iv) “filterSameNamesNotConnected” to retain groups of unconnected nodes containing the same species names. Four types of outputs were implemented on ProKlust: (i) “maxCliques,” the maximal clique is the largest subset of nodes in which each node is directly connected to every other node in the subset; (ii) “components” that contains the isolated nodes or groups formed of complete graphs; (iii) “graph,” an igraph object graph, that can be further handled by the user; and (iv) the “plot,” where the final graph could be promptly visualized with forceNetwork function from the “networkD3” R package (Allaire et al., 2017).

The data generated in the previous topic was clustered with ProKlust to extract groups of genospecies. For both pyANI and FastANI identity matrices, the cut-off criterion chosen was ANI ≥95%. Additionally, the pyANI alignment coverage matrix, representing the fraction of each genome that was aligned, was combined with ANIb, with an arbitrary cut-off point of ≥50%. The data were filtered using the parameters “filterDifferentNamesConnected” and “filterSameNamesNotConnected”.

### Quality Check

For quality check, miComplete (Hugoson et al., 2019) was employed to infer weighted completeness and redundancy of genomes using the precalculated weights associated with the inbuilt marker sets “Bact105.” In miComplete, completeness is calculated based on the presence/absence of a set of marker genes.

Additionally, we analyzed the 16S rRNA gene copies present on our genome set. The 16S rRNA genes were collected from RNA sequences by genomic FASTA. Barrnap^2^ was employed to predict the location of the 16S rRNA genes in *E. terangae* SEMIA 6460^T^. Taxonomic assignments from order to genus ranks were made for the extracted sequences with the IdTaxa function available via the “DECIPHER” v2.14.0 R package (Murali et al., 2018) using the SILVA SSU r138 trained classifier (Yilmaz et al., 2014; link to the full license:^3^).

To guarantee the identity of the genomes present on the RefSeq database, we choose to further analyze the 16S rRNA sequences extracted in a subset of genomes that were selected based on the results from the clustering step or/and were assigned to different genera using the SILVA SSU r138 trained classifier. To provide reliable comparisons, we removed seven sequences with ≤400 nucleotides. The 16S rRNA reference sequences determined by Sanger method from type strains were then retrieved according to the sequence accessions provided on LPSN – List of Prokaryotic names with Standing in Nomenclature (as available at^4^). An additional 16S rRNA sequence for *Methylobacterium organophilum* ATCC 27886^T^ (NR_041027) sequenced by Kato et al. (2005) was added as a reference. The 16S rRNA for “*Bartonella mastomydis*” (KY555064) was retrieved from Dahmani et al. (2018). We performed a profile-to-profile alignment considering RNA secondary structure using “AlignSeqs” function from “DECIPHER.” The “seqinr” v.3.6.1 R package (Charif and Lobry, 2007) was employed to compute pairwise distances from aligned sequences with no gaps.

### Summary of the Study Design

**FIGURE 1:**
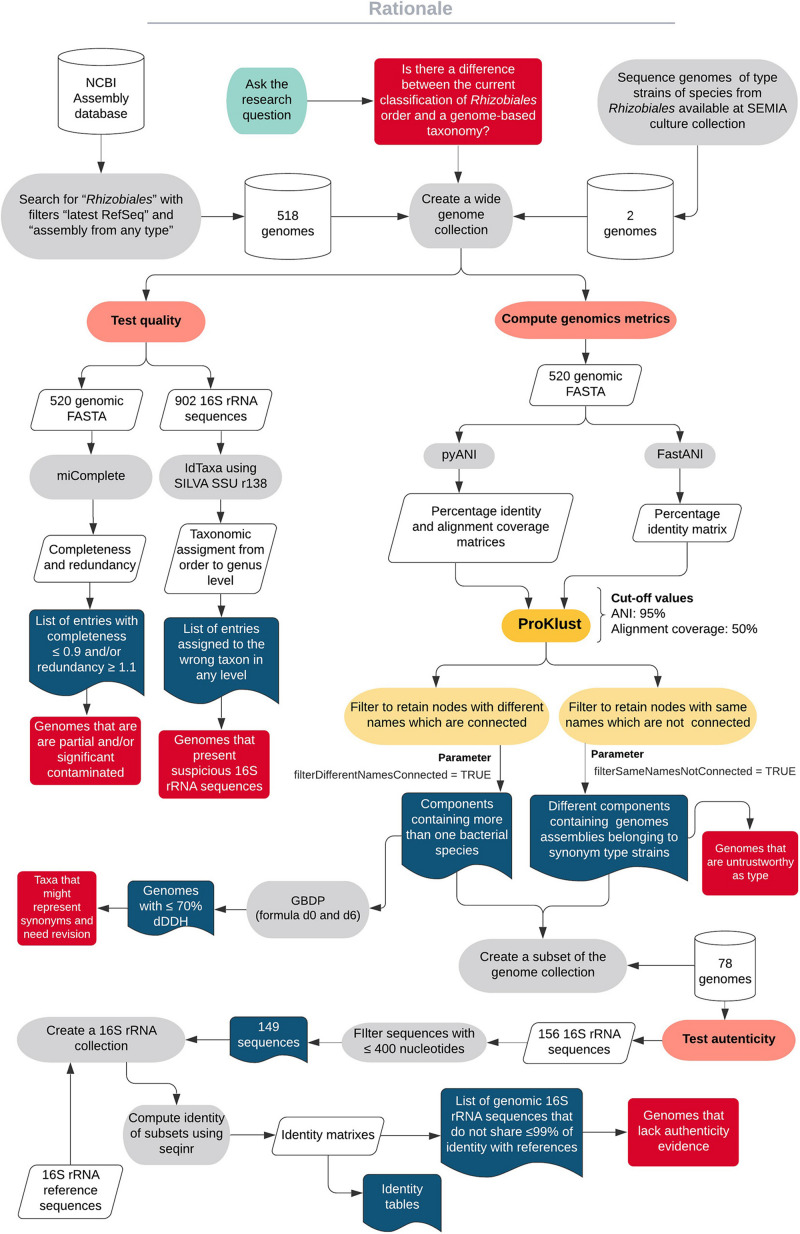
A diagram showing the workflow of the study.

In this work, we first checked the general quality of 520 genomes obtained from the type strains of species from the order *Rhizobiales*. To obtain genomic groups with high identity, the values computed with ANIb and FastANI were clustered using ProKlust. An additional authenticity check was performed to support our proposals of changes in the actual taxonomic classification. To apply Wayne et al. (1987) recommendations, we also computed dDDH using GGDC formula *d0* and *d6* among closely related strains. A diagram capturing the steps performed in this work is shown in Figure 1.

### Data and Code Availability

The genomic sequence generated for *E. terangae* SEMIA 6460^T^ can be found according to the following identifiers: 2838074704 (IMG Taxon OID, Integrated Microbial Genomes platform), Ga0394399 (GOLD Id, Genomes Online Database), and PRJNA581033 (NCBI BioProject Accession). The genomic sequence generated for *R. gallicum* SEMIA 4085^T^ can be found according to the RefSeq accession GCF_013004495.1. The genomes retrieved from NCBI can be found using the RefSeq accessions provided in Supplementary Table 1. ProKlust is available at https://github.com/camilagazolla/ProKlust.

## Results

### Quality Analysis of the Genome Collection

Quality-related statistics obtained from the genome collection are presented in Supplementary Figure 1 and Table 1. The genomes presented a range of 1.44–10.11 Mb in length, with an average weighted completeness and redundancy parameters of 0.9814 and 1.026, respectively. From the 520 genomes present in the set, 12 were detected with weighted completeness ≤0.9 and/or weighted redundancy ≥1.1 (Table 1). This low sequence quality must be considered by taxonomists dealing with these specific accessions.

**TABLE 1:** Genomes detected with high contamination and/or low completeness.

Assembly accession	Refseq category	Organism name	Length (MB)	Contigs	Present markers	Completeness	Redundancy
GCF_000421945.1	NA	*Agrobacterium radiobacter* DSM 30147^T^	7.18	612	70	0.7077	1.6195
GCF_001187535.1	Representative	*Rhizobium ecuadorense* CNPSO 671^T^	7.38	1891	103	0.9813	1.1221
GCF_001641635.1	Representative	*Bradyrhizobium centrolobii* BR 10245^T^	10.11	133	104	0.9814	1.1206
GCF_001723295.1	Representative	*Methyloceanibacter marginalis* R-67177^T^	3.00	470	95	0.8782	1.0293
GCF_001723305.1	Representative	*Methyloceanibacter superfactus* R-67175^T^	3.10	38	98	0.8961	1.0245
GCF_002759055.1	NA	*Methylobacterium frigidaeris* IER25-16^T^	6.40	1670	96	0.893	1.0415
GCF_002866925.1	Representative	*Cohaesibacter celericrescens* H1304^T^	5.02	59	95	0.9684	1.1036
GCF_002930635.1	Representative	*Kaistia algarum* LYH11^T^	6.21	598	104	0.9814	1.1927
GCF_003024595.1	Representative	*Mesorhizobium plurifarium* LMG 11892^T^	4.42	35	105	1	1.1446
GCF_003024615.1	NA	*Mesorhizobium loti* LMG 6125^T^	4.88	52	103	0.9979	1.1806
GCF_003049685.1	NA	*Ochrobactrum pituitosum* CCUG 50899^T^	5.52	10	104	0.9814	1.1275
GCF_902162175.1	NA	*Bartonella saheliensis* 077^T^	2.26	131	90	0.8198	1.1681

*NA, not available.*

We also analyzed the taxonomic assignments of the 16S rRNA genes present in the genome sequences. A total of 902 genes were found among 513 genomes, varying from 1 to 13 copies per genome. We then compared the taxonomic assignments of these copies using SILVA SSU r138 (Yilmaz et al., 2014) to those of the genomes. A total of 715 gene copies were correctly assigned to the genus, 160 gene copies were not taxonomically assigned, and 28 copies were assigned to suspicious taxa (Supplementary Table 2). After the removal of sequences belonging to recently described genera that were absent in the classifier, a total of 17 genomes remained assigned to suspicious copies of the 16S rRNA (Table 2). These were further evaluated to determine if they represented misclassified genome sequences.

**TABLE 2:** 16S rRNA copies extracted from genomes and incorrectly taxonomically assigned.

Name	Access	16S rRNA gene locus tag	SILVA SSU r138		
			
			Order	Family	Genus
*Aquabacter cavernae* Sn-9-2^T^	GCF_003993795.1	EJJ38_RS13400	*Rhizobiales*	*Xanthobacteraceae*	*Xanthobacter*
*Aquabacter spiritensis* DSM 9035 ^T^	GCF_004346185.1	EDC64_RS23195	*Rhizobiales*	*Xanthobacteraceae*	NA
“*Mesorhizobium composti*” CC-YTH430^T^	GCF_004801285.1	E6C48_RS19465	*Rhizobiales*	*Rhizobiaceae*	*Pseudaminobacter*
*Mesorhizobium loti* LMG 6125^T^	GCF_003024615.1	C7U62_RS19230	*Rhizobiales*	*Rhizobiaceae*	*Ensifer*
*Mesorhizobium plurifarium* LMG 11892^T^	GCF_003024595.1	C7U60_RS18680	*Caulobacterales*	*Caulobacteraceae*	*Brevundimonas*
		C7U60_RS19135; C7U60_RS21155	*Rhizobiales*	*Rhizobiaceae*	*Ensifer*
		C7U60_RS18920	NA	NA	NA
*Methylobacterium crusticola* MIMD6^T^	GCF_003574465.1	DT057_RS35310	*Burkholderiales*	*Oxalobacteraceae*	*Massilia*
		DT057_RS35040	*Rhizobiales*	*Beijerinckiaceae*	NA
		DT057_RS35290	*Burkholderiales*	NA	NA
		DT057_RS05830; DT057_RS35200	*Rhizobiales*	NA	NA
		DT057_RS35260	NA	NA	NA
*Methylobacterium frigidaeris* IER25-16^T^	GCF_002759055.1	CS379_RS09215	*Bacillales*	*Bacillaceae*	*Bacillus*
*Mongoliimonas terrestris* MIMtkB18^T^	GCF_001927285.1	BUQ68_RS19420	*Rhizobiales*	*Pleomorphomonadaceae*	*Chthonobacter*
*Oharaeibacter diazotrophicus* DSM 102969^T^	GCF_004362745.1	EDD54_RS17735; EDD54_RS20245	*Rhizobiales*	*Pleomorphomonadaceae*	*Chthonobacter*
*Oharaeibacter diazotrophicus* SM30^T^	GCF_011317485.1	GRZ53_RS14600; GRZ53_RS22395	*Rhizobiales*	*Pleomorphomonadaceae*	*Chthonobacter*
*Rhizobium marinum* MGL06^T^	GCF_000705355.1	EO99_RS0125160	*Rhizobiales*	*Rhizobiaceae*	*Pseudorhizobium*
*Rhizobium vignae* CCBAU 05176^T^	GCF_000732195.1	GQ59_RS30420	*Bacteroidales*	*Prevotellaceae*	*Prevotella*
		GQ59_RS30195	*Rhizobiales*	*Rhizobiaceae*	*Neorhizobium*
		GQ59_RS30200	*Rhizobiales*	*Rhizobiaceae*	NA

*Taxonomic assignments using IdTaxa and reference dataset SILVA SSU r138.*

The 16S rRNA genes extracted from *Aquabacter cavernae* Sn-9-2^T^ and *A. spiritensis* DSM 9035^T^ genomes were both assigned to Xanthobacteraceae. However, these sequences shared a high identity of 99.8 and 100%, respectively, with the 16S rRNA gene references MF958452 and FR733686 (Supplementary Table 3). *Xanthobacter autotrophicus* DSM 432^T^ and *X. tagetidis* ATCC 700314^T^ were reported by Duo et al. (2019) as the nearest phylogenetic neighbors of *A. cavernae* Sn-9-2^T^. The unexpected affiliation of the *A. spiritensis* DSM 9035^T^ 16S rRNA gene sequence within the family Xanthobacteraceae was also reported previously by Yarza et al. (2013). Recently, Hördt et al. (2020) proposed including *Aquabacter* into Xanthobacteraceae. The authors reported that *Aquabacter*, *Xanthobacter*, and *Azorhizobium* formed a highly supported clade in a GBDP *d3* tree and that those genera are difficult to discern as currently circumscribed with 16S rRNA gene analyses. Given that the genome sequences from all the type strains of this genus are not yet available, it remains to be elucidated if *A. cavernae* and *A. spiritensis* represent species of *Xanthobacter*.

“*Mesorhizobium composti*” was proposed by Lin et al. (2019), although the name is not validly published. The assembly employed here was based upon the WGS accession given for the type strain CC-YTH430^T^, and the 16S rRNA sequence extracted from the genome and the reference KX988315 presented 100% identity. In a 16S rRNA gene tree, “*M. composti*” CC-YTH430^T^ was reported to form a cluster with *Mesorhizobium* and *Pseudaminobacter* species (Lin et al., 2019), which could explain its wrong assignment to *Pseudaminobacter* in SILVA SSU r138 (Table 2). Besides, several *Mesorhizobium* species have been described as intermixed with *Pseudaminobacter* in 16S rRNA gene (Lin et al., 2019) and GBDP *d5* phylogenies Hördt et al. (2020). Taken into consideration, the genome available for “*M. composti*” CC-YTH430^T^ seems to be authentic.

*Methylobacterium crusticola* MIMD6^T^ was recently described by Jia et al. (2020) isolated from biological soil crusts. The assembly employed here holds the WGS accession given in the *M. crusticola* description. The *M. crusticola* MIMD6^T^ genome presents six 16S rRNA sequences, of which only the locus tag DT057_RS35040 assigned to Beijerinckiaceae shared a high identity of 99.1% with the reference KT346425 sequence, while the identity of the remaining five sequences ranged from 56.1 to 75.8%. In a core-proteome dendrogram constructed using the neighbor-joining method (Supplementary Figure 2), *M. crusticola* MIMD6^T^ was placed along other *Methylobacterium* strains instead of the ones belonging to Beijerinckiaceae. Considering this, the *M. crusticola* MIMD6^T^ genome is authentic but wrongly designated in SILVA SSU r138 classifier. It remains to be elucidated if those suspiciously assigned copies represent contamination by foreign DNA or even a HGT event. Similarly, *Methylobacterium frigidaeris* IER25-16^T^ presented only one 16S rRNA sequence, and it was assigned to *Bacillus* (Table 2). The WGS accession provided from its description corresponded to the genome employed here (Lee and Jeon, 2018), and despite that, it presented only 86.2% of identity with the reference (KY864396). In the core-proteome dendrogram (Supplementary Figure 2), *M. frigidaeris* IER25-16^T^ was also found along with other *Methylobacterium* instead of *Bacillus* strains. Considering this, the 16S rRNA copy of *M. frigidaeris* IER25-16^T^ is more likely to represent a contamination or HGT event than a reliable taxonomic marker.

The family Pleomorphomonadaceae currently comprises the genera *Chthonobacter*, *Hartmannibacter*, *Methylobrevis*, *Mongoliimonas*, *Oharaeibacter*, and *Pleomorphomonas* (Hördt et al., 2020). The 16S rRNA genes extracted from *Mongoliimonas terrestris* MIMtkB18^T^ and two assemblies belonging to *Oharaeibacter diazotrophicus* (DSM 102969^T^ and SM30^T^) were assigned to the *Chthonobacter* genus. However, the WGS for *M. terrestris* MIMtkB18^T^ genome given by Xi et al. (2017) is comprised within the assembly employed here. Also, the 16S rRNA gene present in the genome is identical to the reference (KP993300), confirming its authenticity. Regarding *O. diazotrophicus*, the four 16S rRNA sequences extracted from its assemblies shared 99.9–100% of identity with the reference for *O. diazotrophicus* SM30^T^ (LC153750; Lv et al., 2017), confirming their authenticity. Remarkably, the 16S rRNA genes of *M. terrestris* and *O. diazotrophicus* share a 16S rRNA sequence identity of 98.4–98.3% and 97.3–97.9%, respectively, with the type species of *Chthonobacter*, *C. albigriseus* ED7^T^ (KP289282). Once the genome of *C. albigriseus* ED7^T^ becomes available, the placement of *Mongoliimonas*, *Oharaeibacter*, and *Chthonobacter* as separate genera should be reevaluated.

The *Rhizobium vignae* CCBAU 05176^T^ genome sequence possessed three different 16S rRNA gene copies, which were ultimately assigned to *Prevotella*, *Neorhizobium*, and an unidentified genus of Rhizobiaceae. The 16S rRNA copy which was assigned to *Neorhizobium* (GQ59_RS30195) presented a high identity of 99.9% with the reference GU128881 given on the *R. vignae* description (Ren et al., 2011), while the remaining copies shared less than 76% identity. Considering this, it seems that GCF_000732195.1 represents an authentic *R. vignae* CCBAU 05176^T^ genome, but contamination or HGT occurred. Recently, Hördt et al. (2020) proposed that *R. vignae* should be assigned to *Neorhizobium* because *R. vignae* was placed as a sister group of *Neorhizobium galegae* (Mousavi et al., 2014) with strong support in a GBDP *d5* tree.

The genomes attributed to *Mesorhizobium plurifarium* LMG 11892^T^ and *Mesorhizobium loti* LMG 6125^T^ indeed represented unauthentic genome sequences. The *Rhizobium marinum* MGL06^T^ is a synonym of *Pseudorhizobium pelagicum*. These issues are explored further in the next section below.

### Genospecies Cluster Detection with ProKlust

**FIGURE 2:**
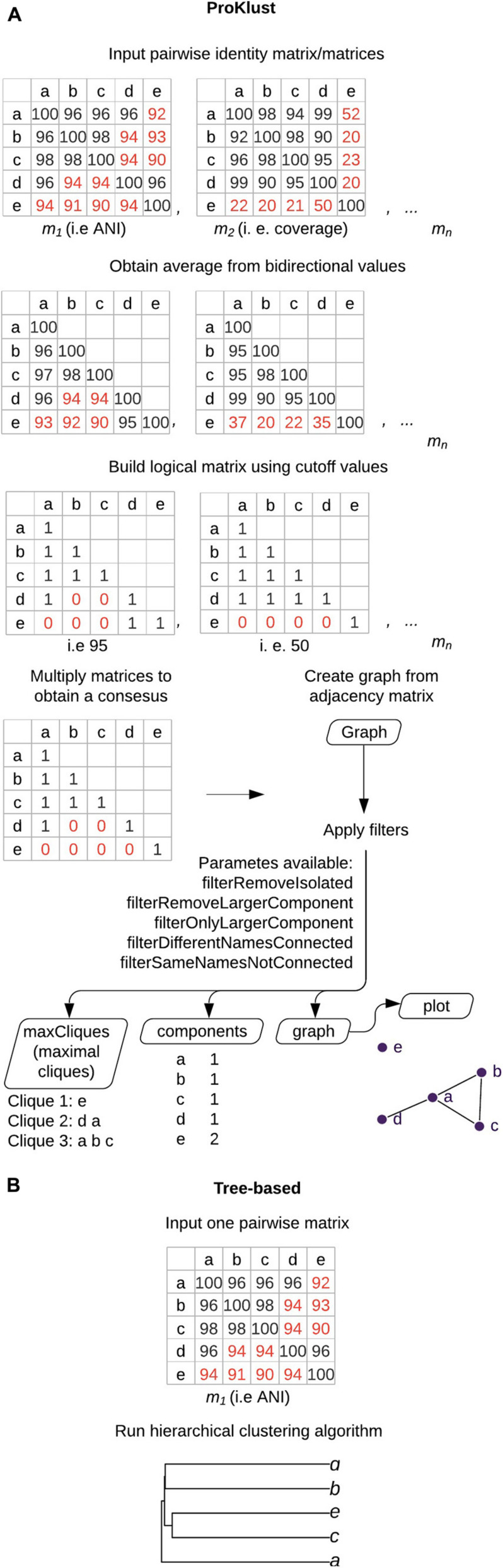
Graph-based clustering using ProKlust compared to hierarchical clustering. **(A)** The average of each pair from the pairwise input. matrix/matrices is/are obtained. A Boolean matrix/matrices is/are obtained according to the cut-off values chosen by the user. If more than one matrix is used as input, the final generated matrix is obtained by multiplying the elements of the matrices. A graph is formed by connecting the nodes which present the positive values. In this example, nodes correspond to genomes and edges correspond to ANI ≥95% with coverage alignment ≥50%. The data could be filtered to retain components containing more than one species name or unconnected nodes containing the same species names. In addition, filters to remove isolated nodes (“filterRemoveIsolated”) or the largest component (“filterOnlyLargerComponent”) are also available. The tool generates four types of outputs: (i) the maximal cliques on “maxCliques,” which is the largest subset of nodes in which each node is directly connected to every other node in the subset i.e., all the possible species groups that could be delimited in the graph, which could result in groups having genomes in common; (ii) “components” that contains the isolated nodes or groups formed of complete graphs; (iii) “graph,” an igraph object graph, that can be further handled by the user; and (iv) the “plot,” where the final graph could be visualized. **(B)** Overview of the hierarchical-based clustering approach. These approaches return tree-shaped diagrams with non-overlapping clusters.

Here, we describe ProKlust, an R package for the downstream analysis of large identity matrices using maximal cliques enumeration (Figure 2). ProKlust is open source and not computationally intensive. Due to its flexibility, ProKlust could be employed to analyze any identity/similarity matrix, such as barcoding gene identity. Additionally, it contains useful filter options to deal with taxonomical data.

A total of 415 components were obtained using ProKlust to analyze the input of 270,400 ANI values computed for the 520-genome set. Components are the groups formed by linking the genomes together according to the chosen criteria, i.e., ≥95% ANI. A genome that is part of a component does not necessarily share ANI values above the established cut-off with all the other genomes of that component, but it must share an ANI value above the cut-off for at least one other genome. Cliques, instead, are formed by genomes that all share ANI values above the chosen criteria. A genome could belong at the same time to different cliques within the same component.

**FIGURE 3:**
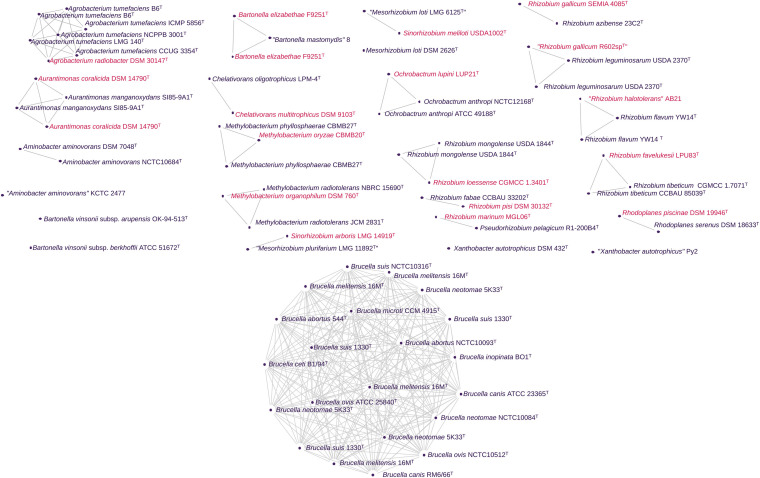
Genomic clusters detected using pairwise ANI values from 520 *Rhizobiales* genomes. Here, only a subset is shown following filtering to (i) retain genomes clusters containing more than one bacterial species name and (ii) the unconnected genomes containing the same species names (synonym strains). In the graph, the clusters have nodes corresponding to genomes and edges corresponding to ANI values above the cut-off for species delineation. In the case of ANIb from pyANI, where an alignment coverage matrix was also generated, the edges additionally correspond to a reliable alignment of between the set of genomes. The same graph structure was obtained using FastANI values. Different colors were used whenever possible to indicate different species names.

All components detected were congruent between the ANIb and FastANI methods. Employing a filter step to retain genomes clusters containing more than one bacterial species name, we were able to easily identify genomes clusters containing heterotypic synonyms on our genome set (Figure 3). Some of these heterotypic synonyms had already been identified by other authors (Table 3). We discuss some of these proposals in addition to the heterotypic synonyms identified. Additionally, we found strains incorrectly assigned as members of the same inspecting the clustering behavior of “supposedly” synonym type strains.

**TABLE 3:** Heterotypic synonyms found here that have already been detected by other authors.

Species 1	Species 2	Basis	Proposition	References
*Agrobacterium tumefaciens*	*Agrobacterium radiobacter*	Numerical taxonomic analysis showing that 24 strains of *A. radiobacter* and *A. tumefaciens* composed a cluster	*A. tumefaciens* is a later heterotypic synonym	Holmes and Roberts (1981)
*Mabikibacter ruber*	*Notoacmeibacter marinus*	dDDH (79.8%) and OrthoANIu (97.8%)	*M. ruber* is a later heterotypic synonym	Huang and Lai (2020)
*Ochrobactrum lupini*	*Ochrobactrum anthropi*	ANIb (97.55%), ANIm (98.25%), gANI (97.99%), OrthoANI (97.94%) and dDDH (83.9%)	*O. lupini* is a later heterotypic synonym	Volpiano et al. (2019)
*Brucella ceti*	*Brucella melitensis*	dDDH (97.8%)	*B. ceti* is a later heterotypic synonym	Hördt et al. (2020)
*Brucella inopinata*	*Brucella melitensis*	dDDH (81.2%)	*B. inopinata* is a later heterotypic synonym	
*Brucella microti*	*Brucella melitensis*	dDDH (99.1%)	*B. microti* is a later heterotypic synonym	
*Brucella vulpis*	*Brucella melitensis*	dDDH (80.5%)	*B. vulpis* is a later heterotypic synonym	
*Methylobacterium phyllosphaerae*	*Methylobacterium oryzae*	dDDH (90.3%)	*M. phyllosphaerae* is a later heterotypic synonym	
*Methylobacterium radiotolerans*	*Methylobacterium organophilum*	dDDH (92.2%)	*M. organophilum* is a later heterotypic synonym	
*Rhizobium marinum*	*Pseudorhizobium pelagicum*	dDDH (76.3%)	New subspecies of *R. marinum* from *P. pelagicum* (*R. marinum* subsp. *pelagicum*)	
*Rhizobium mongolense*	*Rhizobium loessense*	dDDH (70.0%)	New subspecies of *R. mongolense* from *R. loessense* (*R. mongolense* subsp. *loessense*)	

The minimal standards for the use of genome data for the taxonomy of prokaryotes recommend the utilization of 16S rRNA sequences to confirm the authenticity of genome data (Chun et al., 2018). Thus, the 16S rRNA genes found in the 78 genomes presented in Figure 3 were also examined to ensure their authenticity. When the 16S rRNA sequences shared ≥99% identity, it confirmed their assignments to the same operational taxonomic unit (Stackebrandt and Ebers, 2006; Kim et al., 2014). The 16S rRNA distance matrices for each taxon can be found in Supplementary Table 3. The dDDHs using formulas *d0* and *d6* were also computed (Supplementary Table 4) considering the proposal of two measures of genetic relatedness to set the boundary for prokaryotic species established by Wayne et al. (1987).

The genomic clusters formed by *Rhizobium favelukesii* LPU83^T^ and *Rhizobium tibeticum* CCBAU 85039^T^ and CGMCC 1.7071^T^ assemblies possessed an ANI value of 95.9% and a dDDH up to 64.7%. Similarly, the genomes of *R. flavum* YW14^T^ and “*Rhizobium halotolerans*” AB21 shared 95.8% of ANIb and 65.4% of dDDH. These strains should remain classified in separate species according to Wayne et al. (1987).

#### *Aurantimonas manganoxydans* as a Later Heterotypic Synonym of *Aurantimonas coralicida*

*Aurantimonas manganoxydans* SI85-9A1^T^ and “*Aurantimonas litoralis*” HTCC 2156^T^ are described as Mn(II)-oxidizing bacteria isolated from the oxic/anoxic interface of a stratified Canadian fjord and the surface waters of the Oregon coast, respectively (Anderson et al., 2009). Despite sharing nearly identical 16S rRNA gene sequences with the previously described *A. coralicida* WP1^T^ (Denner et al., 2003), the measured DDH similarity between *A. manganoxydans* SI85-9A1^T^ and “*A. litoralis*” HTCC 2156^T^ with WP1^T^ was only 21.8 and 9.45%, respectively.

*Aurantimonas coralicida* DSM 14790^T^ and *A. manganoxydans* SI85-9A1^T^ each have two genome assemblies in the RefSeq database, while a genome sequence for an “*A. litoralis*” type strain is still not available. We found a total of four and two 16S rRNA gene copies in the *A. manganoxydans* SI85-9A1^T^ and *A. coralicida* DSM 14790^T^ genomes, respectively, which shared a high identity with the reference sequences (AJ786361 and AJ786360). Also, these genomes share 95.25–95.24% of ANIb and at least 78.7% of dDDH, greatly differing from the genome relatedness values reported by Anderson et al. (2009).

Thus, *A. manganoxydans* corrig. Anderson et al. (2011) should be considered a later heterotypic synonym of *A. coralicida* Denner et al. (2003) considering the bacterial species threshold of 95% of ANIb originally proposed by Goris et al. (2007).

#### *Chelativorans multitrophicus* and *Chelativorans oligotrophicus* Represent Members of a Single Species

Doronina et al. (2010) proposed the genus *Chelativorans* to accommodate *C. multitrophicus* DSM 9103^T^ isolated from samples taken from industrial wastewater treatment plants and *C. oligotrophicus* LPM-4^T^, isolated from sewage sludge. The two EDTA-degrading strains shared a high similarity of 99.3% between their 16S rRNA gene sequences.

We found that the *C. multitrophicus* DSM 9103^T^ and *C. oligotrophicus* LPM-4^T^ genomes shared 99.86% of ANIb and at least 98.9% of dDDH. Additionally, the 16S rRNA gene sequences extracted from them presented ≥99% identity to the references EF457243 and EF457242, respectively.

Considering these findings, *C. multitrophicus* Doronina et al. 2010 and *C. oligotrophicus* Doronina et al. 2010 represent members of a single species. The former name should be retained because it represents the type species of *Chelativorans* genus.

#### *“Mesorhizobium loti* LMG 6125^T^” (GCF_003024615.1), *“Mesorhizobium plurifarium* LMG 11892^T^” (GCF_003024595.1), and *Methylobacterium organophilum* DSM 760^T^ (GCF_003096615.1) Genomes Should Not Be Used as Reference Points in Taxonomy

After Frank’s (1889) description of *Rhizobium* genus, Jordan (1984) described *R. meliloti*, *R. loti*, and three biovars of *R. leguminosarum* (uniting the former species of *R. leguminosarum*, *R. phaseoli*, and *R. trifolii*). Subsequently, Chen et al. (1988) proposed creation of a separate genus, *Sinorhizobium*, to include the previously described *R. fredii* (Scholla and Elkan, 1984). *Rhizobium meliloti* was later transferred to *Sinorhizobium* (de Lajudie et al., 1994), and *S. arboris* and *S. kostiense* were proposed as new *Sinorhizobium* species (Nick et al., 1999). Jarvis et al. (1997) proposed the genus *Mesorhizobium* for encompassing *R. loti* and *R. huakuii*, after observing that the fatty acid profiles, additional physiological characteristics, and the 16S rRNA genes from these species were distinct from those of members of the genera *Agrobacterium*, *Rhizobium*, and *Sinorhizobium*. Later, de Lajudie et al. (1998) described *M. plurifarium* ORS 1032^T^, isolated from root nodules of *Acacia* species.

Other significant revisions have been made by Willems et al. (2003). These authors utilized several characterization methods, including 16S rRNA and *recA* sequence analyses, and showed that *Ensifer* and *Sinorhizobium* formed a single group in neighbor-joining dendrograms, leading to the conclusion that *Ensifer* and *Sinorhizobium* were synonyms and a proposal that the name *Sinorhizobium* should be preferred to *Ensifer*. However, Young (2003) considered that *Ensifer* has priority over *Sinorhizobium* because it was validly published earlier.

Up to date, there are two deposits in RefSeq for genomes of the *M. loti* type strains: “LMG 6125^T^” (GCF_003024615.1) and DSM 2626^T^ (GCF_003148495.1), both listed as the type strains. However, we found that both genomes unexpectedly share only 74.6% of ANIb and 14.1% of dDDH, indicating that they do not represent the genome sequences of the same species. The “*M. loti* LMG 6125^T^” genome shared 62.5 (*d0*) to 70.1% (*d6*) of dDDH and 99.5% of ANIb with *Ensifer meliloti* USDA1002^T^, which suggested that “*M. loti* LMG 6125^T^” was a strain of *E. meliloti*. The 16S rRNA gene sequence extracted from “*M. loti* LMG 6125^T^” present only 97.6% of identity with the reference (AB680660). On the other hand, the *M. loti* DSM 2626^T^ genome shared only 74.6% of ANIb with *E. meliloti* USDA1002^T^. The 16S rRNA sequences extracted from this second *M. loti* genome presented ≥99% of identity with the reference. In conclusion, “LMG 6125^T^” (GCF_003024615.1) represents an unauthentic genome sequence for the type strain of *M. loti*. The genome of *M. loti* DSM 2626^T^ (GCF_003148495.1) should be used as a reference for this species.

Similarly, “*M. plurifarium* LMG 11892^T^” genome (GCF_003024595.1) shared an ANI of 99.6% and up to 65.6% of dDDH with the genome of *E. arboris* LMG 14919^T^. None of the three 16S rRNA gene copies found in the “*M. plurifarium* LMG 11892^T^” genome presented sufficient identity with the reference sequence (AB681835) to confirm its identity (Supplementary Table 3).

“*Mesorhizobium plurifarium* LMG 11892^T^” genome accession GCF_003024595.1 and “*M. loti* LMG 6125^T^” genome accession GCF_003024615.1 shared high values of 1.15 and 1.18 for weighted redundancy, respectively (Table 1). Moreover, the 16S rRNA gene copies extracted from these genomes were assigned to suspicious taxa (Table 2). Researchers must utilize these assemblies with caution considering the inconsistencies found here. This observation is especially important for the accession GCF_003024595.1, once it is categorized as the representative genome for *M. plurifarium* on RefSeq database.

The genus *Methylobacterium* was first proposed by Patt et al. (1976), and it was defined by the type species *M. organophilum*, a Gram-stain-negative, methane-utilizing bacterium. Thereafter, Green and Bousfield (1983) demonstrated that *M. organophilum* was phenotypically highly similar to the pink-pigmented, facultatively methylotrophic bacteria that do not utilize methane. Consequently, methane assimilation was omitted as an essential feature in the emended description of the genus. As a result, *Pseudomonas rhodos* Heumann (1962), renamed *Methylobacterium rhodinum*; *Pseudomonas mesophilica* Austin and Goodfellow (1979), renamed *Methylobacterium mesophilicum*; and *Pseudomonas radiora* Ito and Iizuka (1971), renamed *Methylobacterium radiotolerans*, were placed in the emended genus *Methylobacterium*. More recently, based on 16S rRNA gene and multi-locus sequence analyses, genomic, and phenotypic data, Green and Ardley (2018) proposed the *Methylorubrum* genus to accommodate the 11 species previously classified in *Methylobacterium*.

There are two deposits on RefSeq for genomes of *M. radiotolerans* type strains: NBRC 15690^T^ (GCF_007991055.1) and JCM 2831^T^ (GCF_000019725.1). Both genomes share ANIb of 99% and at least 84.4% of dDDH with the “M. *organophilum* DSM 760^T^” genome (GCF_003096615.1), thus indicating that the three genomes belong to the same species. The eight 16S rRNA gene copies from *M. radiotolerans* assemblies possess ≥99% identity with the reference (D32227), confirming their authenticity. However, the 16S rRNA gene from the “*M. organophilum* DSM 760^T^” genome shared a low identity of only 96.1% with the reference (AJ400920).

Kato et al. (2005) has already reported that strain DSM 760^T^ was different from the other *M. organophilum* type strain (JCM 2833^T^) in physiological and biochemical characteristics, although their origins were reported to be the same and they were expected to possess the same properties. After conducting an extensive investigation of *M. organophilum* strains, Kato et al. (2005) concluded that DSM 760^T^ had been mislabeled.

We thus recommend that the GCF_003096615.1 assembly should not be used as a reference for the type strain of *M. organophilum* because it would lead to erroneous conclusions. As an example, the recent report of Hördt et al. (2020) used GCF_003096615.1 to propose *M. organophilum* as a later heterotypic synonym of *M. radiotolerans*.

#### *Rhizobium fabae* as a Later Heterotypic Synonym of *Rhizobium pisi*

*Rhizobium leguminosarum* is the nomenclatural type of the genus *Rhizobium* (Frank, 1889), and the type strain, which was isolated from nodules of pea (*Pisum sativum*), has the original designation of 3Hoq18^T^. After the proposal of Jordan (1984) for the reclassification of *R. trifolii* and *R. phaseoli* as two biovars of *R. leguminosarum*, the description of these species of *R. leguminosarum* was included in the second edition of Bergey’s Manual; however, its re-examination was also recommended by Kuykendall et al. (2005). Ramírez-Bahena et al. (2008) in a paper published on 01 November 2008, analyzed the taxonomic status of these species employing DDH and 16S–23S ITS, *rrs*, *recA*, and *atpD* sequence analyses, in addition to phenotypic characteristics. The 16S rRNA gene sequence of *R. leguminosarum* ATCC 10004^T^ (held in the author’s lab since 1990) was compared with the sequence of *R. leguminosarum* USDA 2370^T^, surprisingly sharing only 99.2% of similarity. The authors then compared the sequences of the *recA* and *atpD* genes from strain ATCC 10004^T^ with those of *R. leguminosarum* USDA 2370^T^ and the 16S–23S ITS region of ATCC 10004^T^ with the sequence of *R. leguminosarum* LMG 14904^T^. The results suggested that the strain ATCC 10004^T^ did not belong to the same species of USDA 2370^T^ and LMG 14904^T^. According to the information recorded from culture collections and molecular analyses from 3Hoq18^T^ and additional *R. leguminosarum* type strains, concluded that the ATCC collection distributed different strains with the same accession number. The results obtained from sequence analysis confirmed that the strain ATCC 10004^T^ received in 1,990 was identical to strains DSM 30132 and NCIMB 11478 and was different from strains LMG 14904^T^ and USDA 2370^T^ (which were identical to each other). Also, the strain that was being provided in 2008 by the ATCC under the designation of ATCC 10004^T^ was identical to strains LMG 14904^T^ and USDA 2370^T^. Since strain USDA 2370^T^ was the original deposit corresponding to strain 3Hoq18^T^, it retained the name *R. leguminosarum*. The strain DSM 30132 = NCIMB 11478 (old strain ATCC 10004^T^, incorrectly distributed) shared DDH of 57% with *R. leguminosarum* USDA 2370^T^ and was thus described as *R. pisi*. Finally, regarding the decision about the status of the names *R. trifolii* and *R. phaseoli*, Ramírez-Bahena et al. (2008) suggested that *R. trifolii* should be considered as a later synonym of *R. leguminosarum*.

Later, on 01 December 2008, *R. fabae* was described by Tian et al. (2008). The type strain CCBAU 33202^T^ was isolated from root nodules of *Vicia faba*. According to the 16S rRNA gene analysis, the closest relative of *R. fabae* CCBAU 33202^T^ was reported to be *Rhizobium etli* CFN42^T^ (99.5% similarity), followed by “*R. leguminosarum* bv. *trifolii*” T24 (99.3%), “*R. leguminosarum* bv. *viciae*” USDA2370^T^ (99.1%), and “*R. leguminosarum* bv. *phaseoli*” USDA 2671 (99.1%). The DDH value described from the comparison of *R. fabae* CCBAU 33202^T^ with *R. etli* CFN 42^T^ and strains of the three biovars of *R. leguminosarum* were 19 and 14–43%, respectively.

Here, the genomes of *R. fabae* CCBAU 33202^T^ and *R. pisi* DSM 30132^T^ shared 97.5% of ANIb and at least 87.8% of dDDH. The 16S rRNA gene sequences extracted from them shared high identity with the reference sequences (DQ835306 and AY509899). Consequently, we propose that *R. fabae* Tian et al. 2008 should be considered as a later heterotypic synonym of *R. pisi* Ramírez-Bahena et al. 2008.

#### *Rhizobium azibense* as a Later Heterotypic Synonym of *R. gallicum*. the Genome Accession GCF_013004495.1 of Strain SEMIA 4085^T^ Should Be Used as a Reference for *R. gallicum* Instead of Genome Accession GCF_000373025.1 of strain R-602 sp^T^

*Rhizobium gallicum* was described by Amarger et al. (1997). The type strain R-602 sp^T^ had been isolated from root nodules of field-grown *Phaseolus vulgaris* sampled in France (Geniaux et al., 1993).

We found that the genome available on RefSeq for *R. gallicum* R-602 sp^T^ (GCF_000373025.1) shared an ANIb ≥98% and a dDDH ≥79.2% with the two genomic assemblies available for *R. leguminosarum* USDA 2370^T^. However, the 16S rRNA sequences extracted from R-602 sp^T^ genome shared only 98.6% of identity with the reference (AF008130), suggesting that the assembly was misclassified.

The genome for another representative of the *R. gallicum* type strain (SEMIA 4085^T^) was sequenced in this study. The 16S rRNA sequence extracted from this genome shared 99.8% identity to the reference AF008130, confirming that it was correctly identified. Moreover, SEMIA 4085^T^ shared 80.3–80.4% of ANIb and 21.2–22.3% of dDDH with the R-602 sp^T^ genome and the type strains of *R. leguminosarum*. Thus, the genome of strain SEMIA 4085^T^ (GCF_013004495.1) was different from those of R-602 sp^T^ (GCF_000373025.1) and *R. leguminosarum* USDA 2370^T^ (GCF_002008365.1 and GCF_003058385.1). According to these findings, we recommend that the genome accession of SEMIA 4085^T^ should be used as a reference for *R. gallicum* species. The genome GCF_000373025.1 is not from the genuine type strain R-602 sp^T^ used for the *R. gallicum* species description.

We also detected that *R. gallicum* SEMIA 4085^T^ shared 99.2% of ANIb and at least 83.3% of dDDH with *R. azibense* 23C2^T^. Importantly, the genome of *R. azibense* 23C2^T^ also contained a 16S rRNA sequence that possessed a high identity of 99.13% with the reference sequence (JN624691) and, thus, appeared to be correctly identified. *R. azibense* was described by Mnasri et al. (2014) as representing a genomic group closely related to *R. gallicum* isolated from root nodules of *P. vulgaris*. Considering the genome relatedness found here, *R. azibense* Mnasri et al. 2014 should be considered as a later heterotypic synonym of *R. gallicum* Amarger et al. 1997.

#### *Rhizobium loessense* and *Rhizobium mongolense*

*Rhizobium loessense* was described by Wei et al. (2003) based upon the type strain CCBAU 7190B^T^ (= CGMCC 1.3401^T^) isolated from *Astragalus complanatus* nodules. The 16S rRNA sequence similarities between *R. loessense* CCBAU 7190B^T^ and the most closely related strains described, *Rhizobium galegae* HAMBI 540^T^ and *Rhizobium huautlense* SO2^T^, were 96.8 and 97.5 %, respectively. The DDH similarity between *R. galegae* HAMBI 540^T^ and *R. huautlense* SO2^T^ with *R. loessense* CCBAU 7190B^T^, were reported to be 40.1 and 9.3%, respectively.

*Rhizobium mongolense* USDA 1844^T^ was isolated from *Medicago ruthenica* nodules as described by Van Berkum et al. (1998). Although Wei et al. (2003) cited previous reports where *R. galegae* and *R. huautlense* were grouped with *R. mongolense*, and *R. gallicum* (Wang et al., 1998; Peng et al., 2002), the type-strains from these species were not included in DDH experiments.

Here, *R. loessense* CCBAU 7190B^T^ and two genomes assemblies from *R. mongolense* USDA 1844^T^ share 96.03% of ANIb and 66.3% (C.I. 62.9 – 69.5%, formula *d6*) to 63.3 (C.I. 59.6 – 66.9%, formula *d0*) of dDDH. All the four 16S rRNA sequences extracted from *R. mongolense* assemblies and the one sequence extracted from *R. loessense* CCBAU 7190B^T^ presented high identities with the reference sequences (U89817 and AF364069) confirming their authenticity.

To be coherent with Wayne et al. (1987), those strains should be placed in separate species. However, this case is open for alternative interpretations. Hördt et al. (2020) obtained 70% of dDDH between *R. mongolense* USDA 1844^T^ and *R. loessense* CGMCC 1.3401^T^ and proposed the new subspecies *R. mongolense* subsp. *loessense* from *R. loessense* based on a threshold of <79% for subspecies according to Meier-Kolthoff et al. (2014).

#### *Rhodoplanes piscinae* as a Later Heterotypic Synonym of *Rhodoplanes serenus*

*Rhodoplanes piscinae* was described with the type strain JA266^T^ isolated from a surface water sample from a freshwater fishpond (Chakravarthy et al., 2012). According to the 16S rRNA gene phylogeny, *R. piscinae* JA266^T^ (= DSM 19946^T^) was closely related to *R. serenus* TUT3530^T^ (= DSM 18633^T^) isolated from pond water and described by Okamura et al. (2009). To differentiate *R. piscinae* JA266^T^ from its closest relative, Chakravarthy et al. (2012) conducted a DDH experiment between both strains, which yielded a value of less than 65%.

In this work, *R. piscinae* DSM 19946^T^ and *R. serenus* DSM 18633^T^ genomes shared 97.6% of ANIb and 88.5% of dDDH. The 16S rRNA gene sequences extracted from these genomes were compatible with the reference sequences (LC178579 and AB087717) and confirmed the identity of the assemblies. We thus propose that *R. piscinae* Chakravarthy et al. 2012 should be considered as a later heterotypic synonym of *Rhodoplanes serenus* Okamura et al. 2009.

#### *“Bartonella mastomydis”* as a Later Heterotypic Synonym of *Bartonella elizabethae*

“*Bartonella mastomydis*” was proposed by Dahmani et al. (2018), with the type strain 008^T^ being isolated from *Mastomys erythroleucus* rodents. Based on a phylogeny reconstructed from concatenated *gltA*, *rpoB*, 16S RNA, and *ftsZ* sequences, *B. elizabethae* F9251^T^ (Brenner et al., 1993) was recognized as the closest relative to strain “*B. mastomydis*” 008^T^, with a dDDH value of 60.3 ± 2.8%.

Here, “*B. mastomydis*” 008^T^ and two genome assemblies from *B. elizabethae* F9251^T^ were found to share an ANIb of 95.1% and at least 89.5% of dDDH. The 16S rRNA sequences extracted from “*B. mastomydis*” 008^T^ and *B. elizabethae* F9251^T^ genomes shared identity values (≥99%) with reference sequences KY555064 and L01260, respectively, confirming their identity. These results suggest that “*B. mastomydis*” represents a synonym of *B. elizabethae* (Daly et al. 1993) Brenner et al. 1993.

#### *Bartonella vinsonii* subsp. *arupensis* and *Bartonella vinsonii* subsp. *berkhoffii* Represent Members of Different Species

*Bartonella vinsonii* was proposed by Brenner et al. (1993), with the type strain ATCC VR-152^T^ isolated from voles by Baker, 1946. Later, Kordick et al. (1996) isolated the strain 93-C01^T^ from the blood of a dog with valvular endocarditis. According to the 16S rRNA gene analysis, 93-C01^T^ was closely related to the type strain of *B. vinsonii*. According to DDH tests, 93-C01^T^ and *B. vinsonii* ATCC VR-152^T^ genomes shared 81% (hybridization at 55°C) to 70% (hybridization at 70°C) of DDH and 5°C of ΔTm. Kordick et al. (1996) then proposed *B. vinsonii* subsp. *berkhoffii* to accommodate 93-C01^T^, considering that: (i) a second isolate (G7464) was significantly more closely related to 93-C01^T^ than both were related to *B. vinsonii*; (ii) 93-C01^T^ and G7464 shared a unique insertion in their 16S rRNAs; and (iii) both were isolated from dogs.

In 1999, Welch et al. (1999) compared an isolate from human blood culture (OK 94-513^T^) to *Bartonella* species. They reported that its reciprocal DNA relatedness to *B. vinsonii* subsp. *vinsonii* and *B. vinsonii* subsp. *berkhoffii* was 65 to 85% at 55°C. Based on these and other results, proposed creating a third subspecies within *B. vinsonii*, *B. vinsonii* subsp. *arupensis* with the type strain OK 94-513^T^.

We found that the genomes of *B. vinsonii* subsp. *berkhoffii* ATCC 51672^T^ and *B. vinsonii* subsp. *arupensis* OK-94-513^T^ were not within the same genome cluster, sharing ANIb values of only 91.3%. The dDDH values present between them were 74.5 (*d0*) and 81.5% (*d6*). To be consistent with the Wayne et al. (1987) recommendation, these strains should be placed in separate species. The 16S rRNA sequences extracted from *B. vinsonii* subsp. *arupensis* and subsp. *berkhoffii* genomes presented high identity with the reference sequences of HF558389 and L35052, respectively, confirming their identity. The taxonomic status of the three *B. vinsonii* subspecies may need to be revised after sequencing the type-strain of *B. vinsonii* subsp. *vinsonii*.

#### “False Type Strain” Genome Sequences

So far, we have focused on classification and authenticity errors. Now we deal with the third type of error where genomes are erroneously assigned as type strains in the databases. These errors were discovered by the identification of clusters containing more than one described bacterial species as well as different genomes assemblies supposedly belonging to the same strains that were not clustered together.

The genomes of *X. autotrophicus* DSM 432^T^ (GCF_005871085.1) and “*X. autotrophicus*” Py2 (GCF_000017645.1) presented only 91% of ANIb and up to 57.6% of dDDH, consequently, the strains were not found forming a cluster. In 1986, Van Ginkel and De Bont, 1986 isolated bacterial strains from soil and water samples that were able to grow in an atmosphere of 5% alkene in the air. Based on physiological, morphological, and GC content data, the yellow-pigmented strain “*X. autotrophicus*” Py2 was assigned to the genus *Xanthobacter*. Meanwhile, the original type strain of *X. autotrophicus* was isolated from a black pool sludge 8 years before (Wiegel et al., 1978). Considering this, “*X. autotrophicus*” Py2 was incorrectly identified as a type strain in the RefSeq database.

Genomes of *Aminobacter aminovorans* DSM 7048^T^ (GCF_004341645.1) and NCTC10684^T^ (GCF_900445235.1) shared ANIb and dDDH of 100%. A third type strain, “*A. aminovorans*” KCTC 2477^T^ (GCF_001605015.1), was not clustered with the other two strains. DSM 7048^T^ and NCTC 10684^T^ indeed represented *A. aminovorans* type strain, while KCTC 2477^T^ (= ATCC 29600^T^ = DSM 10368^T^) does not. KCTC 2477^T^ was classified as the type strain of *Chelatobacter heintzii* before being reclassified as *A. aminovorans* (Kämpfer et al., 2002). In the KCTC 2477^T^ genome announcement (Lee S.H. et al., 2016), the strain is wrongly assigned as *A. aminovorans* type strain, which can explain its erroneous assignment in the NCBI metadata.

#### Proposal of *Aminobacter heintzii* comb. nov. Reclassification of *Aminobacter ciceronei* as *Aminobacter heintzii*, and *Aminobacter lissarensis* as *Aminobacter carboxidus*

“*Pseudomonas*” spp. ATCC 29600^T^ was isolated by successive enrichment in a medium with nitrilotriacetate (NTA) from a soil surrounding a dry well that had received septic tank effluent (Tiedje et al., 1973). In a study of NTA-utilizing organisms, Auling et al. (1993) described *Chelatobacter* genus with ATCC 29600^T^ representing *C. heintzii* as the only species. Later, *C. heintzii* was reclassified based on (i) similarities above 99 ± 6% between the 16S rRNA gene sequences from *C. heintzii* DSM 10368^T^ with *A. aminovorans* DSM 7048^T^, *A. aganoensis* DSM 7051^T^, and *A. niigataensis* DSM 7050^T^ (Kämpfer et al., 1999); and (ii) a DDH similarity study that showed that *C. heintzii* DSM 10368^T^ strain shared DDH values of at least 70% with *A. aminovorans* DSM 7048^T^ (Kämpfer et al., 2002).

Here, the *C. heintzii* KCTC 2477^T^ genome shared an ANIb of only 90.8% and a dDHH value up to 65.2% with the two assemblies of *A. aminovorans* DSM 7048^T^ and NCTC 10684^T^. The 16S rRNA gene sequences extracted from *C. heintzii* KCTC 2477^T^ and *A. aminovorans* assemblies were compatible with their reference sequences (LT984904 and AJ011759, respectively) and confirmed their identities. Considering this, *C. heintzii* KCTC 2477^T^ and *A. aminovorans* DSM 7048^T^ represent members of different species.

To identify additional strains belonging to the same species as *C. heintzii* KCTC 2477^T^, 21 genome sequences for *Aminobacter* were obtained, including a recently deposited genome for DSM 10368^T^. They included the genomes for *A. aganoensis* DSM 7051^T^, *A. carboxidus* DSM 1086^T^, *A. ciceronei* DSM 17455^T^ and DSM 15910^T^, *A. lissarensis* DSM 17454^T^, and *A. niigataensis* DSM 7050. The quality of these genomes was checked (Supplementary Table 5), and the comparisons were calculated using ANIb, dDDH, and ProKlust as described before.

**FIGURE 4:**
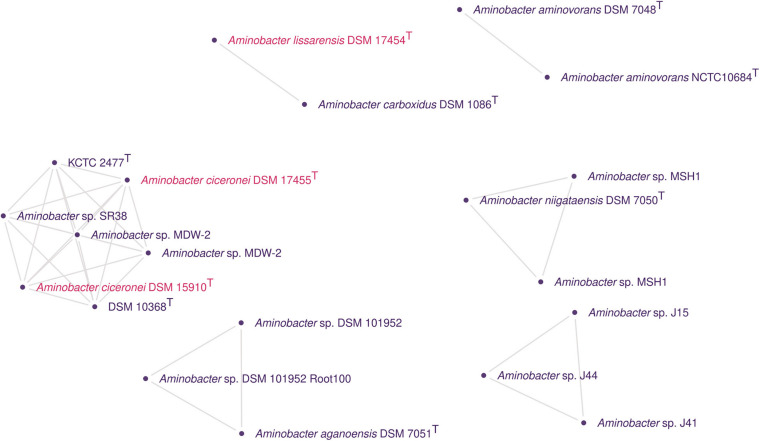
Genomic clusters detected using pairwise ANI values from *Aminobacter* genomes. In the graph, the clusters have nodes corresponding to genomes and edges corresponding to ANI values above the cut-off for species delineation with a reliable alignment of between the genomes. Isolated nodes were removed. Different colors were used whenever possible to indicate different species names.

In addition to the expected *C. heintzii* DSM 10368^T^ genome, the cluster containing *C. heintzii* KCTC 2477^T^ (Figure 4) also included *Aminobacter* sp. SR38, *Aminobacter* sp. MDW-2 (accessions GCF_009674635.1 and GCF_014250155.1), and *A. ciceronei* DSM 17455^T^ (GCF_014138635.1) and DSM 15910^T^ (GCF_014138625.1). Importantly, all the genomes forming this cluster shared ANIb values above 98%. The dDDH calculated (Supplementary Table 6) with formula *d6* between these strains ranged from 74.1 to 100%. All comparisons presented a dDDH average of ≥70% for formula *d0*, except between *A. ciceronei* DSM 17455^T^/DSM 15910^T^ with *Aminobacter* sp. SR38 (69.8, CI: 65.8 – 73.4%), and *C. heintzii* DSM 10368^T^ and *Aminobacter* sp. SR38 (69.9, CI: 66 – 73.5%). The 16S rRNA sequences extracted from *C. heintzii* DSM 10368^T^ and *A. ciceronei* DSM 17455^T^/DSM 15910^T^ genomes were compatible with the reference sequences LT984904 and AF034798, respectively.

All the strains present in the *C. heintzii* KCTC 2477^T^/DSM 10368^T^ cluster were pesticide-/herbicide-degrading bacteria. Rousseaux et al. (2001) isolated *Aminobacter* sp. SR38 (= LR3-3), an atrazine-degrading bacterium, from agricultural French soil with a history of treatment with the herbicide. The nearest neighbor of SR38 was *C. heintzii* (AJ011762) according to the 16S rRNA gene analysis conducted. MDW-2 was isolated from sludge from the wastewater-treating system of a pesticide manufacturer (Zhang et al., 2017). The strain can degrade methomyl completely in biochemical cooperation with *Afipia* sp. MDW-3 (Zhang et al., 2017). MDW−2 was also reported to show the highest similarity with *A. aganoensis* DSM 7051^T^ (AJ011760).

IMB-1^T^ (= DSM 17455^T^ = DSM 15910^T^) was isolated from CH_3_Br-fumigated soil in California. It has been reported to be capable of growth on CH_3_Cl, CH_3_Br, CH_3_I, and methylated amines as sole carbon and energy sources (Miller et al., 1997; Connell Hancock et al., 1998). After, McDonald et al. (2005) proposed the species *A. ciceronei* to accommodate IMB-1^T^ based on DDH comparisons with *A. aminovorans* DSM 7048^T^ (47.7%), *A. aganoensis* DSM 7051^T^ (37.2%), and *A. niigataensis* DSM 7050^T^ (17.9%). In the same paper, *A. lissarensis* was also proposed to accommodate CC495^T^ (= DSM 17454^T^), a strain isolated from unpolluted beech woodland soil in Northern Ireland by enrichment culture using CH_3_Cl (Coulter et al., 1999). *A. lissarensis* CC495^T^ was able to grow on methylamine as sole carbon and energy source, as well as with CH_3_Cl and CH_3_Br with cyanocobalamin supplementation. The DDH analysis of *A. lissarensis* CC495^T^ indicated that it shared 20.8, 49.5, and 31.8% hybridization with *A. aminovorans* DSM 7048^T^, *A. aganoensis* DSM 7051^T^, and *A. niigataensis* DSM 7050^T^, respectively. The authors also reported that replicate DDH experiments between *A. aminovorans* DSM 7048^T^ and strain *A. lissarensis* CC495^T^ gave widely differing values (20.8–72.0%) in reciprocal hybridizations. Here, we found that *A. lissarensis* DSM 17454^T^ shared 98.1% of ANIb and at least 81.7% of dDDH with *A. carboxidus* DSM 1086^T^.

Strain Z-1171^T^ (CIP 105722^T^ = DSM 1086^T^) was isolated from soil in Moscow (Russia) and first described as *Achromobacter carboxydus* (Nozhevnikova and Zavarzin, 1974). Z-1171^T^ was assigned to the physiological group of carboxydobacteria due to its ability to grow aerobically on carbon monoxide as the sole carbon and energy source (Zavarzin and Nozhevnikova, 1977; Meyer and Schlegel, 1983). Meyer et al. (1993) transferred *A. carboxydus* to a new genus *as Carbophilus carboxidus* based on 16S rRNA similarities and phenotypic characteristics. Recently, Hördt et al. (2020) reported a 16S rRNA gene tree where *C. carboxidus* CIP 105722^T^ was nested within *Aminobacter*, leading to the transferring *Carbophilus* to *Aminobacter*.

Considering the genome relatedness found here, we propose *A. heintzii*, comb. nov. as a new combination for *C. heintzii* Auling et al. 1993. We also propose the reclassification of *A. ciceronei* McDonald et al. 2005 as *A. heintzii*. Finally, *A. lissarensis* McDonald et al. 2005 should be considered as a later heterotypic synonym of *A. carboxidus* (Meyer et al. 1994) Hördt et al. 2020.

## Discussion

The results of the OGRI and ProKlust analyses revealed several inaccuracies with the taxonomic scheme with the type strains of species from the order *Rhizobiales*. This came as no surprise considering that the rapid expansion of sequenced bacterial and archaeal genomes in the past decade (Garrity, 2016; Hugenholtz et al., 2016; Yoon et al., 2017a) was accompanied with numerous reclassification and name changes (Sant’Anna et al., 2017; Nouioui et al., 2018; García-López et al., 2019; Volpiano et al., 2019; Hördt et al., 2020). The most likely reason for this is that traditional DDH and ΔTm measurements are more imprecise than the application of ANI and dDDH *in silico* surrogates (Auch et al., 2010b; Meier-Kolthoff et al., 2013).

The genome metrics approach is based on computationally intensive pairwise genomic alignments and calculations, which are a disadvantage in large-scale studies. FastANI uses fast approximate read mapping with Mapmash, based on MinHash alignment identity estimates, being reported to be 50-4608 times faster than ANIb (Jain et al., 2018). We found that the same groups of genospecies in our set of 520 genomes were obtained using both ANIb and FastANI algorithms. To guarantee accuracy and to reduce the computing cost for species delineation, FastANI could be employed to sieve a lower total number of genomes for subsequent alignment and calculation of identity with ANIb and dDDH.

ProKlust demonstrated to be a useful graph-based approach to extract genomic groups from large OGRI matrices, which can also be applied to other identity/similarity matrices. Our tool has the advantage of settable cut-off points, the possibility of multiple matrices entries, besides useful functions to filter and visualize the obtained clusters. Graph-based approaches depend upon finding cliques or completely connected subgraphs utilizing a threshold value. Higher threshold values generally result in a less connected graph and therefore smaller cluster sizes (Jay et al., 2012). To date, we have found two graph-based tools that were useful with OGRI data. The first is the “Genome Clustering” web-based tool from MicroScope (Microbial Genome Annotation and Analysis Platform) described by Vallenet et al. (2009). The interface allows the user to define a set of 3 to 500 genomes from the current 5,033 genomes available at the platform. The user can also add their own data. The platform estimates the genomic similarity using Mash distances, which are reported to be well correlated to ANI, especially in the range of 90–100% (Ondov et al., 2016). To obtain genome clusters, “Genome Clustering” i) connects all nodes using the pairwise Mash distances; ii) removes edges representing distances above 0.06 (which is expected to correspond to 94% of ANI); (iii) removes incomplete or contaminated genomes with CheckM (Parks et al., 2015); and (iv) extracts communities from the network with the Louvain community detection algorithm (Blondel et al., 2008). The second approach that we have found is described by Varghese et al. (2015), which also employs an approach based on MCE to analyze the genome-wide ANI (gANI) metric and AF between the matrices generated from 13,151 genomes. They employed a PERL script that uses the C++ Bron–Kerbosch module to construct maximal cliques using the criteria of a minimum pairwise AF of at least 0.6 and a minimum pairwise gANI of at least 96.5%. However, the script used by the authors was not made available with the publication, and no filter parameter seems to be available.

When dealing with genome assemblies from public databases, taxonomists should be aware of the level of contamination and the presence of misidentified type strains on their genome sets (Salvà-Serra et al., 2019). In our study, a series of erroneous conclusions could have been made if the noteworthy presence of misidentified type strains and unauthentic genomes found in our genome set was not previously detected. The 16S rRNA gene analysis and a careful check on genome associated metadata have proved to be an important sanity check step on wide genome-based taxonomy surveys, which could prevent, for example, the recent report of Hördt et al. (2020) to propose the reclassification of *M. radiotolerans* using comparisons with an unauthentic *M. organophilum* genome.

Genomic and phenotypic approaches have unique contributions to microbial taxonomy, but the integration of both worlds is challenging (Sanford et al., 2021), especially in studies dealing with hundreds of strains. Phenotypic evaluation is important to characterize a taxon of interest, but its accuracy is affected by differential gene expression and interpretation bias (Petti et al., 2005; Vital et al., 2015; Sant’Anna et al., 2017). Nevertheless, an evaluation of phenotypic and morphologic properties of problematic type-strains found here, along with a comparison with the species’ descriptions, would corroborate that these strains are misidentified, but was not within the scope of this study.

The results of this study are considered to contribute to improving the taxonomic classification of species within *Rhizobiales* order. The genome clustering approach and the tool described here are proven as useful to detect species and to provide identity information from large genome sets.

## Taxonomic Consequences

### Description of *Aminobacter heintzii* comb. nov.

Basonym: *Chelatobacter heitzii* Auling et al. 1993.

*Aminobacter heintzii* (hein.tzii, N. L. gen. masc. n. *heintzii* of Heintz’s, named after the chemist W. Heintz, an honor proposed by Auling et al., 1993).

The description for the type strain KCTC 2477^T^ = ATCC 29600^T^ = DSM 10368^T^ is as given for *C. heitzii* (Auling et al., 1993). Phenotypes for strain IMB-1 = DSM 17455 = DSM 15910 are present by Connell Hancock et al. (1998) and McDonald et al. (2005). Strains SR38 = LR3-3 and MDW-2 are characterized by Rousseaux et al. (2001) and Zhang et al. (2017).

## Data Availability Statement

The datasets presented in this study can be found in online repositories. The names of the repository/repositories and accession number(s) can be found in the article/Supplementary Material.

## Author Contributions

CV, FS, and AA elaborated the conception of the study. CV wrote ProKlust, collected and analyzed the data, and wrote the manuscript. JS and AB provided the strain SEMIA 6460^T^. ES and WW provided sequence for SEMIA 4085^T^ and SEMIA 6460^T^ genomes, respectively. FS, AA, JS, AB, BL, LV, WW, ES, and LP revised the manuscript critically. All authors read and approved the final manuscript.

## Conflict of Interest

The authors declare that the research was conducted in the absence of any commercial or financial relationships that could be construed as a potential conflict of interest.

## Footnotes

1
https://github.com/widdowquinn/pyani

2
https://github.com/tseemann/barrnap

3
https://creativecommons.org/licenses/by/4.0/legalcode

4
https://lpsn.dsmz.de
