# Erratum: Genomic Metrics Applied to *Rhizobiales (Hyphomicrobiales)*: Species Reclassification, Identification of Unauthentic Genomes and False Type Strains

**DOI:** 10.3389/fmicb.2021.734850

**Published:** 2021-07-26

**Authors:** Frontiers Production Office

**Affiliations:** Frontiers Media SA, Lausanne, Switzerland

**Keywords:** ANI, dDDH, species-cluster, genome clustering, *Rhizobium*

Due to a production error, the bacterial strain taxonomy designations were incorrectly formatted as numbers and the related citations were also formatted incorrectly. The publisher apologizes for this mistake.

A correction has been applied in all cases.

The original article has been updated.

