# Supplementary material for: Genomic Metrics Applied to Rhizobiales (Hyphomicrobiales): Species Reclassification, Identification of Unauthentic Genomes and False Type Strains
Source: Front Microbiol. 2021 Mar 25;12:614957. doi: 10.3389/fmicb.2021.614957 (PMC8026895; doi:10.3389/fmicb.2021.614957)
Supplement: Supplementary file 1 [file Image_1.PDF]

Supplementary Figures

Genomic metrics applied to *Rhizobiales* (*Hyphomicrobiales*): species reclassification, identification of unauthentic genomes and false type strains

Camila Gazolla Volpiano[1], Fernando Hayashi Sant'Anna[1], Adriana Ambrosini[1], Jackson Freitas Brilhante de São José[2], Anelise Beneduzi[2], William B. Whitman[3], Emanuel Maltempi de Souza[4], Bruno Brito Lisboa[2], Luciano Kayser Vargas[2], Luciane Maria Pereira Passaglia[1*]

[1]Departamento de Genética, Instituto de Biociências, Universidade Federal do Rio Grande do Sul, Porto Alegre, Brazil

[2]Departamento de Diagnóstico e Pesquisa Agropecuária, Secretaria Estadual da Agricultura, Pecuária e Desenvolvimento Rural, Porto Alegre, Brazil

[3]Department of Microbiology, University of Georgia, Athens, GA, United States

[4]Departamento de Bioquímica e Biologia Molecular, Centro Politécnico, Setor de Ciências Biológicas, Universidade Federal do Paraná, Curitiba, Brazil

*** Correspondence:**
Luciane Passaglia
luciane.passaglia@ufrgs.br

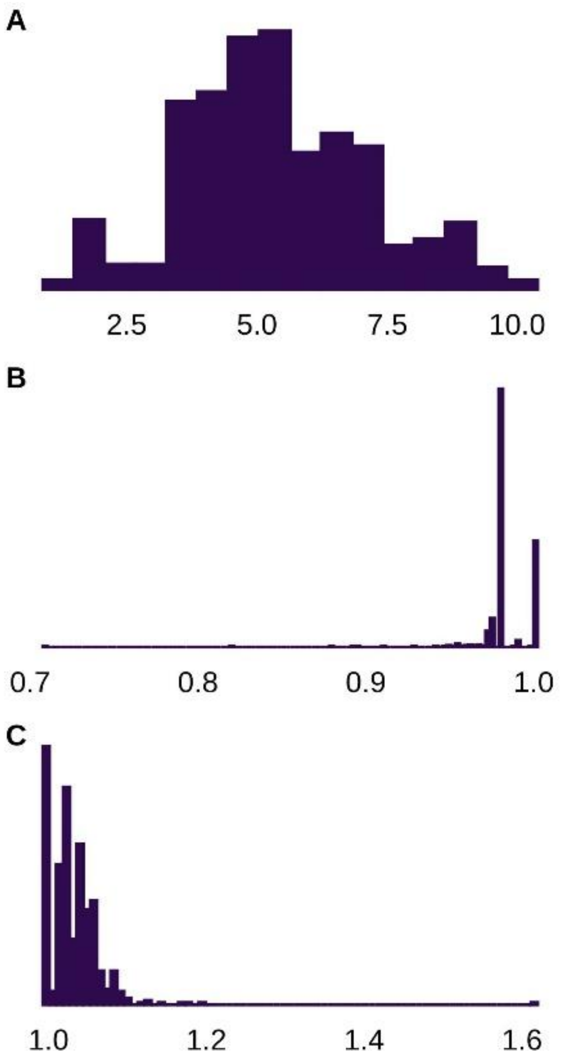

Supplementary Figure 1. Summary of properties of the genome sequences examined in this study. A. Genome length (MB). B. Weighted completeness. C. Weighted redundancy. Summary of data: A. Min. = 1.44, 1st Qu. = 4.19, Median = 5.17, Mean = 5.33, 3rd Qu. = 6.57, Max. = 10.11. B. Min. = 0.7077, 1st Qu. = 0.9814, Median = 0.9814, Mean = 0.9828, 3rd Qu. = 0.9918, Max. = 1.0000. C. Min. = 1.000, 1st Qu. = 1.012, Median = 1.026, Mean = 1.032, 3rd Qu. = 1.047, Max. = 1.619. The redundancy is reported as the fraction duplicated markers of all markers. In similar software, such as CheckM (Parks et al., 2015), this is reported as contamination by percentage. E.g. 1.026 is equivalent to 2.6% of contamination in CheckM.

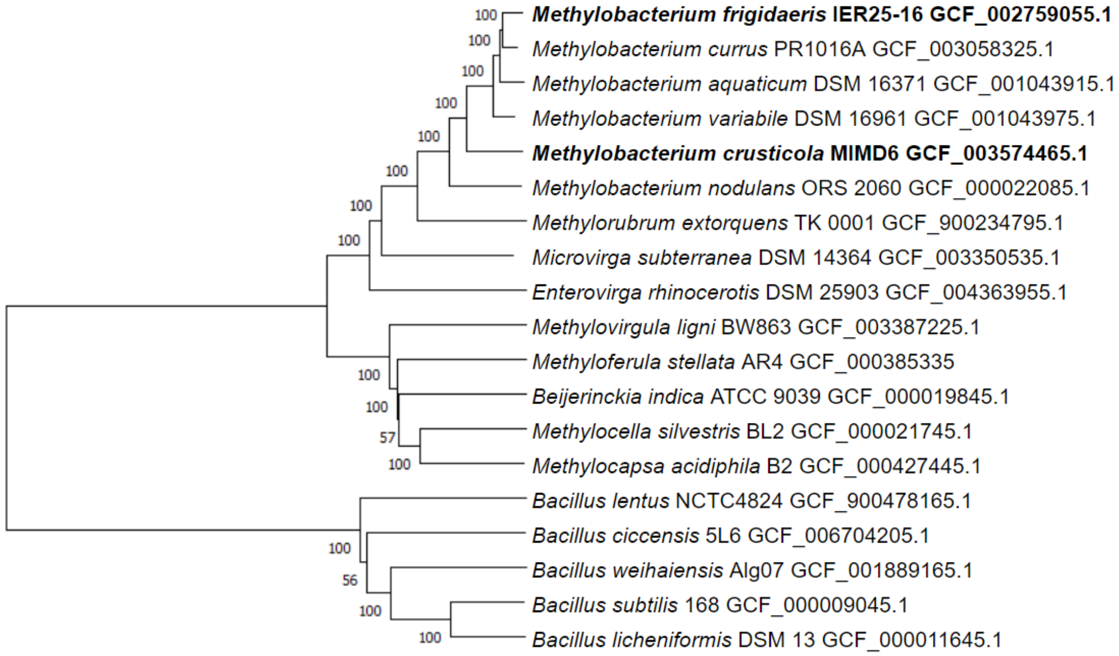

Supplementary Figure 2. Core-proteome dendrogram constructed using the neighbor-joining method as described in Sant'Anna et al. (2017). The ortholog protein groups from genomes were defined using bidirectional best hits algorithm implemented in GET_HOMOLOGUES version 26022020 (Contreras-Moreira and Vinuesa, 2013), using minimum BLAST searches. Clusters containing inparalogs were excluded. Each of the 209 single-copy proteins was aligned with MUSCLE (Edgar, 2004) and concatenated with Phyutility (Smith and Dunn, 2008). The dendrogram was built using the neighbor-joining (Saitou and Nei, 1987) method with the JJT (Jones–Taylor–Thornton) substitution model (Jones et al., 1992). The rate variation among sites was modelled with a gamma distribution (shape parameter=5). The percentage of replicate trees in which the associated taxa clustered together was evaluated with a bootstrap test (1000 replicates). The analysis was conducted in MEGA X (Kumar et al., 2018).

Contreras-Moreira, B. and Vinuesa, P. (2013) GET_HOMOLOGUES, a versatile software package for scalable and robust microbial pangenome analysis. Appl. Environ. Microbiol., 79, 7696-701. doi: 10.1128/AEM.02411-13.

Edgar, R. C. (2004) MUSCLE: multiple sequence alignment with high accuracy and high throughput. Nucleic Acids Res., 32, 1792-1797. doi: 10.1093/nar/gkh340.

Jones, D. T., Taylor, W. R., Thornton, J. M. (1992) The rapid generation of mutation data matrices from protein sequences. Comput. Appl. Biosci., 8, 275–282. doi: 10.1093/bioinformatics/8.3.275.

Kumar, S., Stecher, G., Li, M., Knyaz, C., Tamura, K. (2018) MEGA X: molecular evolutionary genetics analysis across computing platforms. Mol. Biol. Evol. 35, 1547-1549. doi: 10.1093/molbev/msy096.

Saitou, N. and Nei, M. (1987) The neighbor-joining method: a new method for reconstructing phylogenetic trees. Mol. Biol. Evol., 4, 406–425. doi: 10.1093/oxfordjournals.molbev.a040454.

Sant'anna, F. H., Ambrosini, A., De Souza, R., De Carvalho Fernandes, G., Bach, E. et al. (2017). Reclassification of *Paenibacillus riograndensis* as a genomovar of *Paenibacillus sonchi*: genome-based metrics improve bacterial taxonomic classification. Front. Microbiol. 8, 1849. doi: 10.3389/fmicb.2017.01849.

Smith, S. A. and Dunn, C. W. (2008) Phyutility: a phyloinformatics tool for trees, alignments and molecular data. Bioinformatics, 24, 715–716. doi: 10.1093/bioinformatics/btm619.
